# Access to and affordability of healthcare for TB patients in China: issues and challenges

**DOI:** 10.1186/s40249-016-0096-y

**Published:** 2016-01-29

**Authors:** Shenglan Tang, Lixia Wang, Hong Wang, Daniel P. Chin

**Affiliations:** Duke Global Health Institute, Duke University, Durham, USA; Global Health Research Center, Duke Kunshan University, Kunshan, China; National Center for TB control and prevention, China CDC, No 155 Changbai Road, Changping District, Beijing 102206 People’s Republic of China; Bill & Melinda Gates Foundation, Seattle, Washington USA; Bill & Melinda Gates Foundation, Beijing office, Beijing, China

**Keywords:** China-Gates TB project, TB control, Healthcare financing, China

## Abstract

This paper introduces the background, aim and objectives of the project entitled “China—the Gates Foundation Collaboration on TB Control in China” that has been underway for many years. It also summarizes the key findings of the nine papers included in this special issue, which used data from the baseline survey of Phase II of the project. Data were collected from the survey of TB and MDR-TB patients, from designated hospitals, health insurance agencies and the routine health information systems, as well as key informant interviews and focus group discussions with relevant key stakeholders. Key issues discussed in this series of papers include the uses of TB services and anti-TB medicines and their determining factors related to socio-economic and health systems development; expenditures on TB care and the financial burden incurred on TB patients; and the impact of health insurance schemes implemented in China on financial protection.

**Electronic supplementary material:**

The online version of this article (doi:10.1186/s40249-016-0096-y) contains supplementary material, which is available to authorized users.

## Multilingual abstract

Please see Additional file 1 for translations of the abstract into the six official working languages of the United Nations.

## Background

In 2013, the World Health Organization (WHO) estimated that China had the second highest burden of both tuberculosis (TB) and multi-drug resistant tuberculosis (MDR-TB) in the world [1]. According to the National TB Epidemiology Survey conducted in 2010, the prevalence of pulmonary TB was 459 per 100,000. TB prevalence in rural areas (569 per 100,000) was almost twice of that in urban areas. In the less developed western region, TB prevalence was 695 per 100,000, which was almost three times than the developed eastern region [2].

Although the high prevalence of TB is still a serious concern in many parts of China, the 2010 Epidemiology Survey revealed an impressive 65 % decline in smear-positive pulmonary TB between 1990 and 2010 [2]. This decline could be attributed to the nationwide scale-up of the WHO-recommended DOTS strategy, which was implemented in the public health system through the Center for Disease Control (CDC). The implementation of DOTS shifted TB treatment regimen from hospitals to CDC, and significantly improved TB treatment on a national scale.

Even with the implementation of DOTS, there is still a lot of TB treatment in the hospital system. The poor treatment outcome in the hospital system has been well documented, and it is one of the main drivers of the MDR-TB epidemic. The 2007 National TB Drug Resistance Survey showed that 5.7 % of new patients and 25 % of re-treated patients had MDR-TB [3]. Inappropriate TB treatments and treatment interruption have been recognized as the most important contributing factors of high MDR-TB prevalence in China [3, 4].

According to the national government policy, essential TB care is free of charge. However, studies have identified additional medical costs associated with the diagnosis and treatment of TB, such as the costs for liver-protection drugs and extra tests, which were paid out-of-pocket by the patients [5–7]. The diagnosis and treatment of MDR-TB has not yet been incorporated into China’s National TB Control and Prevention Program. MDR-TB treatment is often expensive, with treatment duration being 24 months or longer. Expenses associated with TB and MDR-TB treatment may be partially covered by health insurance for patients enrolled in one of the health insurance schemes in China. However, patients still have to pay significant amount of deductibles and co-payments. Moreover, Chinese hospitals are mostly financed by service fees, and insurance schemes reimburse on a fee-for-services (FFS) basis, which provide perverse incentives for hospitals to offer more services, contributing to a rapid increase in healthcare costs [8]. With the addition of considerable non-medical costs and indirect costs associated with TB/MDR-TB care, patients often suffer from heavy financial burden, particularly those patients from poor households.

In the context of new round Chinese health system reform towards universal health coverage, the Chinese National Health and Family Planning Commission (then the Ministry of Health), and China Center for Disease Control and Prevention (CDC), with the support from the Bill and Melinda Gates Foundation, has since 2009 been developing an innovative program on TB and MDR-TB prevention and control in six provinces since 2009. This paper firstly outlines the background, aim and objectives of Phase II of the project entitled “China—the Gates Foundation Collaboration on TB Control in China” (thereafter called “China–Gates TB Project”), and then introduces the nine papers in this special issue using data from the baseline survey of the China-Gates project.

### Introduction of the China-Gates project

From 2009 to 2012, the first phase of the “China-Gates project” focused on MDR-TB diagnosis, treatment and financing. During the first phase of the program, China CDC had undertaken individual pilots to improve the diagnosis, and treatment of MDR-TB, as well as the affordability of MDR-TB care. A comprehensive program was developed that comprised of new diagnostic strategies, standardized treatment based on resistance testing, and collaboration between city hospitals, CDC, and health insurance agencies [9]. The first phase has successfully completed [9]. During the first phase, an important change in TB care and control system took place in China, which was tasks of TB diagnosis and treatment were shifted from CDC to the hospital system [10]. An increasing number of county general hospitals were appointed as TB designated hospitals. Thereafter, the diagnosis and treatment of all TB patients were the responsibilities of the designated hospitals. In addition, funding for TB services will also be mainly provided by health insurance schemes instead of the special fund earmarked for TB from the Ministry of Finance, as it has been with the implementation of DOTS strategies from the 1990s until recently [10]. Therefore, a main challenge in TB care and control that China is facing is how TB services can be effectively financed and delivered in the new system, for instance, how to ensure that the county-level designated hospitals would effectively adopt new TB diagnostic strategies and provide treatment following the national guidelines. The program, with these anticipated changes in China’s national TB control program, decided to tackle these challenges in its second phase starting in 2012. The second phase of the project expanded the scope of work by including all TB patients, and integrating the financing of TB care into China’s health insurance schemes, in line with the ongoing reform of public hospitals. The aim of the second phase of the project was to establish comprehensive TB control models which can be scaled up by the National TB Prevention and Control Program in the future. Specifically, key objectives included: (1) developing comprehensive TB control models for effective diagnosis, treatment and management of TB patients using innovative tools and delivery approaches; (2) exploring financing models and incentive mechanisms for TB care and control, aligning with on-going health system reform in China; and (3) demonstrating and verifying the effectiveness and feasibility of the comprehensive TB control models in selected cities. A comprehensive evaluation of these interventions is planned for late 2015. Results on the impacts of these interventions will be disseminated afterwards.

One main component of the second phase was to develop and implement new financing models of TB and MDR-TB care and control, among others, in the three cities (Zhenjiang City, Jiangsu Province; Yichang City, Hubei Province; and Hanzhong City, Shaanxi Province), which are geographically located in the Eastern, Middle and Western Region of China. The China CDC and the three municipal governments, with the strong support from the Gates Foundation and international experts, have reached the agreement on the financing of TB/MDR-TB care, in collaboration with the New Cooperative Medical Scheme in the rural (NCMS), and Ministry of Human Resource and Social Security (MOHRSS) which is responsible for the two urban health insurance schemes – the urban employee basic health insurance (UEBHI) and the urban resident basic health insurance (URBHI), as well as the Medical Financial Assistance (MFA) schemes managed by the Ministry of Civil Affairs, after several rounds of consultations and negotiations. Two major reforms have been put in place in the three program sites: (1) All the health insurance schemes have agreed to increase the reimbursement rate to 90 % for MDR-TB related inpatient and outpatient services, and increase the reimbursement rate to 70–80 % for TB related outpatient and inpatient services. In addition, MDR-TB patients and TB patients living in poverty or recognized as vulnerable based on local policies will also receive financial subsidies for food and transportations provided by the county Bureau of Civil Affairs. (2) A case-based payment method would be used to reimburse the TB designated hospitals at the prefectural and county levels for their provision of TB/MDR-TB care, in order to motivate hospitals to provide standard treatment and contain cost. The two reforms have since 2014 been implemented, though the implementation plans differ slightly across project cities and counties.

A baseline survey was undertaken to understand pre-intervention practice in TB/MDR-TB financing in the three project cities, and implications for equity, efficiency and effectiveness in relation to the provision, accessibility and utilization of TB/MDR-TB related services. The aim of the baseline survey were: (1) to better understand the current situation of TB/MDR-TB care financing and provider payment mechanism in the three cities, and (2) to generate baseline data to evaluate the effects of the new financing models for TB/MDR-TB care and case-based payment methods in terms of equity and efficiency of TB care; and (3) to examine the provision and quality of TB care and control in different project sites. It was also intended to generate baseline data that can be used to compare TB/MDR-TB care before and after the above interventions in each project site and evaluate their effects/impacts at the end of the project implementation.

The baseline survey was conducted in the three project cities (Zhenjiang, Yichang and Hanzhong) in 2013. Methodological details of the baseline survey are reported in the papers of this special issue [11–18]. Briefly, in each city, the Gross Domestic Products (GDP) per capita in 2012 was used to classify the counties’ level of socio-economic development into low-, middle- and high-group. One county from each development category was randomly selected in each city, resulting in a total of nine counties. Data collection employed a combination of quantitative and qualitative methods including:

1. Patient survey with TB and MDR-TB patients;
2. Key informant interviews and focus group discussions with key stakeholders including policy-makers, service providers, users, and health insurance managers;
3. Institution-based survey with health bureaus, designated hospital, CDC, health insurance agencies and civil affairs bureaus in city and county levels, and township /community health centers using self-administered questionnaires; Field observations and inspections;
4. Routine data on TB suspect reporting, diagnosis, treatment, and case management;
5. Policy document collected for review and analysis.

Researchers from four Chinese universities (the Central China University of Science and Technology, Nanjing Medical University, Shandong University, and Xi’an Jiaotong University), Duke University/Duke Kunshan University, and China CDC have participated in the data collection and analyses, with support from the Gates Foundation and provincial and municipal CDCs in these project sites. This special issue is a collection of papers presenting key findings emanating from the baseline survey. The papers were jointly produced by the researchers from the collaborative organizations and finalized at the two writing workshops hosted by Central China University of Science and Technology in Wuhan and China CDC in Beijing, respectively in 2014.

### The introduction of papers

Nine papers included in this special issue aim to discuss issues related to the utilization of TB services and anti-TB medicines and their influencing factors of socio-economic and health systems development, medical and non-medical expenses of TB care and their financial burdens placed on TB patients, and the impacts of health insurance schemes implemented in China on provision of financial protection. The last paper of this collection tackles the increasing challenge of MDR-TB in China.

Over-provision and under-provision of TB services in the Chinese hospitals co-exists at present. Hu and his colleagues reported high admission rates in the project sites of their paper, entitled “Factors that affect TB patient admission rate and TB patient cost” and identified three main factors that were closely associated with TB patient admission rates: (1) economic incentives which encouraged hospitals to generate revenues from TB services, (2) misunderstanding of infectious disease control that promoted hospitalization, and (3) health insurance policies that potentially induce the utilization of TB services in designated hospitals [11]. The identification of these factors provides evidence to develop relevant policies aimed to reduce unnecessary inpatient services for TB patients. In the article entitled “Anti-tuberculosis drug use in China: a retrospective study”, Huang et al. assessed inpatient medical records from 12 sampled hospitals (three prefectural hospitals and nine county hospitals) to judge the rational use of anti-TB drugs based on WHO’s TB treatment guidelines [12]. The examination of TB treatment regimens reveals that more than half of the inpatients might be treated with irrational regimens, and the anti-TB drugs were more appropriately prescribed in the prefecture-level hospitals than in the county general hospitals. In addition, the overuse of second-line anti-TB drugs (SLD), particularly in the county general hospitals, was a serious problem. All these reflect that the effectiveness of TB care is in jeopardy in many study hospitals, as inadequate regiments would result in poor treatment outcomes, while over-provision of services would harm the efficiency of service delivery. Hence, these two papers highlight the importance to improve TB financing strategies such as the provider payment policies; to enhance professional and health education programs for TB treatment and control at the community level; and to strengthen clinical governance by improving the adherence of the hospitals to the clinical guidelines in order to achieve acceptable effectiveness and efficiency of TB care delivery.

The over-provision of TB services often results in increased expenditures, and thus likely to incur greater financial burden on health insurance funds and the families of the TB patients. This special issue includes four papers examining the expenditures on TB services and financial burden of TB care on patients. The first paper by Jia and her colleagues examined actual practices and costs of TB services observed in the study hospitals, in light of the so-called “free” essential TB care in China [13]. The paper reported substantial medical expenditures for completing TB treatment, particularly among patients who received both outpatient and inpatient care. The second paper by Zhou and his associates examined the incidence, intensity and determinants of catastrophic health expenditure (CHE), using two thresholds proposed by WHO: annual expenditure on TB care (a) exceeds 10 % of total household income, and (b) exceeds 40 % of the household’s non-food expenditure [14]. The paper revealed that the average out-of-pocket payment for TB care exceeded both thresholds. According to Zhou et al., some of the significant determinants of CHE were age, household size, employment status, health insurance status, and patient income as a percentage of total household income. The paper “Are free anti-TB drugs enough? An empirical study from three cities in China,” written by Chen et al., shed light on the relationship between patient treatment adherence and the relative economic burden experienced by TB patients [15]. The perceived economic burden of TB care erected barriers for low-income TB patients seeking diagnosis and treatment; however, the authors argued that non-medical expenditures played a greater role in noncompliance than previously thought. Li and his colleagues examined non-medical expenditures on TB care including costs on transport, accommodation and nutritional supplements, and found 20 % of TB patients reported catastrophic expenditure on non-medical expenditures [16]. Economic burden as a deterrent to pursuing medical care comes in multiple forms. The key findings from these two papers have brought up a serious concern on equity in financing of TB care, and particularly its disproportionate economic impact on the poor patients.

Two papers included in the special issue intend to assess the impact of health insurance schemes being implemented in China on financial protections for the TB patients. The first paper entitled “Disparity of TB care reimbursement among different health insurance schemes: Evidence from three counties in central China*”* by Pan and her colleagues adopts a comprehensive approach to understand China’s current medical insurance system and evaluate the disparity in TB care reimbursement between the three major insurance schemes – NCMS, UEBHI and URBHI [17]. By comparing the three major schemes, the authors showed that those covered under UEBHI had the highest average reimbursement rates for TB care, while enrollees under the NCMS had the lowest rates, again showing that patients in lower socioeconomic classes were more susceptible to financial hardship due to TB treatment costs. Such a situation is worrying, as a vast majority of TB patients living in the rural areas where only NCMS operates. While Pan et al. focused on all three health insurances schemes in China, Xiang and colleagues focused only on the NCMS and its effect on TB care reimbursement rates for patients in rural China [18]. This study found significant discrepancies between the effective and nominal reimbursement rates for inpatient care, as well as inconsistencies between the rates reported in the patient database and those reported in medical records. In addition, outpatient reimbursement packages were limited and less comprehensive than inpatient packages. A key finding from their study revealed that though it had some limited impact on the severity of CHE, the implementation of the NCMS did not reduce the magnitude of CHE for TB patients. In other words, the implementation of NCMS in rural China, initiated a decade ago, did not provide adequate financial protection to most TB patients.

The last paper of the special issue is “Drug-resistant tuberculosis control in China: progress and challenges” written by Long and her colleagues [19]. It presents the current situation of MDR-TB in China, and analyzes and discusses main factors associated with such a rising challenge the government of China is facing. The authors also proposed strategies that China should take to tackle MDR-TB epidemic in the years to come.

## Conclusions

The survey we have undertaken has generated many interesting and important findings, particularly on financing of TB/MDR-TB care and control in these program sites. Although almost all the patients are covered by three health insurance schemes (NCMS, UEBHI & URBHI), at least two schemes (NCMS and URBHI) fails to provide adequate financial protection for the use of TB outpatient services, as they prioritized the coverage of inpatient care in the service benefit packages. Consequently, the out of pocket payment for TB and MDR-TB outpatient services were considered high, especially for those patients from low income groups. Catastrophic health expenditure (CHE) in TB care seeking was not uncommon among TB patients. In the meantime, only a small number of TB patients enjoyed the benefits offered by MFA schemes, as either the eligible criteria were too strict or the procedures were too complicated. In addition, hospital admission rates for TB patients were high in most of hospitals we surveyed, as the service providers might try to increase revenue through over-provision of services, although the extent varied among different cities and counties as reported in some papers of this special issue. All these findings emanating from the baseline survey are critical, based on which interventions were proposed and are currently implemented to improve equity, efficiency and effectiveness of TB care and control in China.

## Additional file

Additional file 1:
**Multilingual abstracts in the six official working languages of the United Nations.** (PDF 254 kb)

## Abbreviations

CDC: center for disease control and prevention
CHE: catastrophic health expenditure
FFS: fee for services
MDR-TB: multi-drug resistant tuberculosis
MFA: medical financial assistance
NCMS: new cooperative medical scheme
SLD: second-line anti-TB drugs
TB: tuberculosis
UEBHI: Urban Employee basic health insurance
URBHI: Urban residence basic health insurance
