# Supplementary material for: Access to and affordability of healthcare for TB patients in China: issues and challenges
Source: Infect Dis Poverty. 2016 Jan 29;5:10. doi: 10.1186/s40249-016-0096-y (PMC4731945; doi:10.1186/s40249-016-0096-y)
Supplement: Additional file 1: — Multilingual abstracts in the six official working languages of the United Nations. (PDF 254 kb) [file 40249_2016_96_MOESM1_ESM.pdf]

Translation of the abstract into the six official working languages of the United Nations

إتاحة الرعاية الصحية ويسر تكلفتها لمرضى السل في الصين: المشكلات والتحديات

"شينجلان تانج"، "ليكشيا وانج"، "هونج وانج" و"دانيال بي تشين"

الملخص:

هذا البحث بمثابة مقدمة لخلفية وغرض وأهداف المشروع المعنون "التعاون بين الصين ومؤسسة جايتس لمكافحة مرض السل في الصين" الذي يجري منذ عدة أعوام، كما أنه يلخص النتائج الأساسية للأبحاث التسع المتضمنة في هذا العدد الخاص، التي استخدمت بيانات مستمدة من المسح الأساسي للمرحلة الثانية من المشروع. جُمعت تلك البيانات من مسح لمرضى السل (المعروف اختصارًا بـTB) والسل المقاوم للأدوية المتعددة (المعروف اختصارًا بـMDRTB)، من المستشفيات المخصصة ووكالات التأمين الصحي وأنظمة المعلومات الصحية المعتادة، إلى جانب مقابلات مع أهم المجيبين ومناقشات مجموعات بؤرية مع أهم المساهمين ذوي الصلة. أهم المشكلات التي نوقشت في هذه السلسلة من الأبحاث تشمل استخدامات خدمات مرض السل والأدوية المضادة له وعواملها الحاسمة المرتبطة بتنمية الأنظمة الاجتماعية- الاقتصادية والصحية؛ والنفقات المتعلقة برعاية مرض السل والعبء المالي الذي يتكبده مرضى السل؛ وتأثير أنظمة التأمين الصحي المطبقة في الصين على الحماية المالية.

Translated from English version into Arabic by Heba Kandel, through

TRANSLATORS WITHOUT BORDERS

中国结核病人卫生服务可及性和可负担性：问题和挑战

汤胜蓝，王黎霞，汪宏，Daniel P Chin

摘要

中盖结核病防治项目已执行多年，目前正在开展项目二期工作。这篇文章介绍了中盖结核病防治项目的背景，目标和目的。该文也简介了纳入此专刊的 9 篇文章。这些文章均使用了中盖结核病防治项目二期的基线调查数据，包括结核/耐药结核病人问卷调查，结核病定点医院和医疗保险机构的登记数据，以及知情人访谈和焦点组访谈。纳入此专刊的这些文章主要讨论了结核诊疗服务和抗结核药的使用以及与之相关的社会经济和卫生体系因素；结核诊疗服务费用和病人面临的经济负担；以及目前医保政策对减轻病人经济负担的作用和影响。

Translated from English version into Chinese by Tang Shenglan

Accès et abordabilité des soins de santé pour les patients tuberculeux en Chine : problèmes et défis

Shenglan Tang, Lixia Wang, Hong Wang, & Daniel P Chin

Résumé

Le présent article expose le contexte, le but et les objectifs du projet intitulé « Chine : collaboration de la Fondation Gates dans la lutte contre la tuberculose en Chine ». Il résume également les principaux résultats de neuf articles du présent numéro spécial, utilisant des données de l'étude de base de Phase II du projet. Les données ont été recueillies au moyen d'enquêtes sur les patients tuberculeux (y compris des cas de tuberculose MR), auprès des hôpitaux désignés, des caisses d'assurance-maladie et des systèmes d'information sanitaire, ainsi que par des entretiens avec des informateurs haut placés et des discussions en groupe de travail avec les parties prenantes concernées. Les grands problèmes discutés dans cette série d'articles concernent le recours aux services de soins contre la tuberculose et aux médicaments antituberculeux et leurs facteurs déterminants en lien avec le développement socioéconomique et celui des systèmes de santé publique, le coût du traitement de la tuberculose et l'impact des programmes d'assurance-maladie appliqués en Chine en termes de protection financière.

Translated from English version into French by Suzanne Assenat, through

Возможность получения лечения и его ценовая доступность для туберкулезных больных в Китае: проблемы и трудности

Шэнлань Тан (Shenglan Tang), Лися Ван (Lixia Wang), Хун Ван (Hong Wang), Дэниел П. Чин (Daniel P. Chin)

Аннотация

Эта статья знакомит с кратким описанием, целью и задачами проекта «Китай – сотрудничество с Фондом Гейтс по контролю над распространением ТБ в Китае», работы по которому ведутся много лет. В ней также обобщены ключевые результаты исследований, изложенные в девяти статьях, входящих в данный специальный выпуск, в котором используются данные базисного обследования фазы II упомянутого проекта. Данные были получены при обследовании больных ТБ и МЛУ-ТБ, от заранее выделенных для этого больниц, агентств медицинского страхования и обычных систем со здравоохранительной информацией, а также из опросов информантов и дискуссий фокус-групп при участии главных заинтересованных лиц. Основные вопросы, обсужденные в этой серии статей, включают пользование услугами и лекарствами для лечения ТБ, их решающие факторы, связанные с развитием социально-экономических и здравоохранительных систем; расходы на лечение ТБ и денежные трудности, с которым сталкиваются больные ТБ; влияние внедрённых в Китае схем медицинского страхования на финансовую защиту населения.

Translated from English version into Russian by Natalia Potashnik, through

Accesibilidad a la atención médica y posibilidad de afrontar los costos que esta conlleva para los pacientes que padecen tuberculosis en China: problemas y desafíos

Shenglan Tang, Lixia Wang, Hong Wang y Daniel P. Chin

Resumen

En este trabajo se presentan los antecedentes, el propósito y los objetivos del proyecto titulado “China – Colaboración de la Fundación *Gates* para la lucha antituberculosa en China”, el cual viene desarrollándose desde hace muchos años. Además, se resumen las principales conclusiones de los nueve trabajos que se incluyen en este número especial, en los cuales se utilizaron los datos obtenidos de la encuesta inicial de la Fase II del proyecto. Los datos provienen de la encuesta realizada a pacientes que padecen tuberculosis (TB) y tuberculosis multirresistente (MDRTB), de una serie de centros médicos, instituciones de seguro médico y sistemas de información sanitaria de rutina, así como también de entrevistas con informantes clave y discusiones grupales con referentes de la especialidad. Los temas principales que se analizan en esta serie de trabajos incluyen los usos de los servicios de atención médica para los pacientes con tuberculosis, los medicamentos para combatir la enfermedad y los factores determinantes asociados al desarrollo de los sistemas socioeconómicos y sanitarios; los gastos de atención médica de los pacientes con tuberculosis y la carga financiera que implica para estos; y el impacto de los sistemas de seguro médico que se implementan en China en la protección financiera de estos pacientes.

Translated from English version into Spanish by Mónica Algazi, through

TRANSLATORS
WITHOUT BORDERS
